# Robust Security Beamforming for SWIPT-Assisted Relay System with Channel Uncertainty

**DOI:** 10.3390/s22010370

**Published:** 2022-01-04

**Authors:** Ruijie Guo, Chunling Fu, Yong Jin, Zhentao Hu, Lin Zhou

**Affiliations:** 1 School of Artificial Intelligence, Henan University, Zhengzhou 450046, China; guorj@vip.henu.edu.cn (R.G.); jy@henu.edu.cn (Y.J.); hzt@henu.edu.cn (Z.H.); zhoulin@henu.edu.cn (L.Z.); 2 School of Physics and Electronics, Henan University, Kaifeng 475004, China

**Keywords:** physical layer security, energy harvest, robust optimization, SWIPT, worst case optimization

## Abstract

This paper considers the physical layer security (PLS) of a simultaneous wireless information and power transfer (SWIPT) relay communication system composed of a legitimate source–destination pair and some eavesdroppers. Supposing a disturbance of channel status information (CSI) between relay and eavesdroppers in a bounded ellipse, we intend to design a robust beamformer to maximum security rate in the worst case on the constraints of relay energy consumption. To handle this non-convex optimization problem, we introduce a slack variable to transform the original problem into two sub-problems firstly, then an algorithm employing a semidefinite relaxation (SDR) technique and S-procedure is proposed to tackle above two sub-problems. Although our study was conducted in the scene of a direct link among source, destination, and eavesdroppers that is non-existing, we demonstrate that our conclusions can be easily extended to the scene for which a direct link among source, destination and eavesdroppers exist. Numerical simulation results compared with the benchmark scheme are provided to prove the effectiveness and superior performance of our algorithm.

## 1. Introduction

The future Wireless Sensor Network (WSN) or Internet of Things (IoT) network is composed of billions of energy-limited devices. Since it is frequently deployed in harsh environments, those energy-limited devices are usually difficult to charge. How to give a stable energy supply to those energy-limited devices and thus prolong the life cycle of WSNs is a hot research topic today. Simultaneous wireless information and power transfer (SWIPT) have been proposed as a promising technology to provide power as well as to transmit information for wireless devices, thus it can effectively prolong the life of the energy-limited devices [1]. Moreover, since wireless charging techniques are employed, SWIPT may be flexibly used in electronic devices embedded in building structures or human bodies. Therefore, SWIPT technology in WSN has aroused extensive interest [2].

Due to the dynamic topology of mobile WSN and the open nature of wireless channels, information transmission in WSN may be easy to be intercepted and eavesdropped. At present, the security of wireless communication systems is commonly protected by a cryptographic encryption method which is based on multi-layer protocols. However, the complicated encryption system may induce huge computing challenges at base stations and users, while secure transmission of keys is still difficult to be guaranteed [3]. The physical layer security (PLS) technique is proposed to handle the above problem; since it is free from computational burden caused by encryption and decryption and works well even if the eavesdropper has strong computing power, the foreground of PLS in security of WSN is nice [4].

Traditionally, the cooperative relay technique widely used in WSN is employed to achieve spatial diversity gain as well as to expand WSN coverage [5]. PLS of cooperative relay WSN is a noteworthy issue too. More specifically, to improve PLS is to improve the confidentiality rate of the signal received by users. PLS methods of cooperative relay WSN are divided roughly into two categories. The first is the cooperative jammer (CJ) method, in which the relay transmits artificial noise (AN) to interfere with eavesdroppers. In [6], the relay cluster is divided into two parts, one amplifies and forwards the signal, the other produces AN to interfere with eavesdroppers. In [7], the received signal of each relay is divided into two parts, one is used to forward the signal, and the other is used to harvest energy and then use harvested energy to generate AN to interfere to eavesdroppers. However, generating AN may consume additional energy of energy-limited services embedded in the relay as well as interfere with the legitimate receiver. The other way related to PLS in cooperative relay WSN is the secure beamforming. Security rate and computation complexity of some schemes including maximum secure rate and the zero-space beamforming at total power constraints and individual power constraints of the relay are discussed in [8]. Li proposed the secure relay beamforming algorithm based on concave convex optimization at the power constraints of relay transmitter and energy collector in a multi-antenna SWIPT relay network [9]. The joint optimization of beamformer and energy division ratio in a multi-antenna relay network is also proposed [10]. In an energy-limited full-duplex (FD) relay network, time allocation ratio, energy, and information beamformer are jointly optimized to achieve the maximum secrecy rate [11]. Assuming the channel status information (CSI) of eavesdropper and CSI of legitimate are uncorrelated, Yang proposed a zero-space beamforming algorithm to force the confidential signal into zero space of the eavesdropper to enhance information security [12].

Although much work has been done on secure beamforming, the above research is merely carried out by supposing the CSI of the legitimate receiver and eavesdropper fully known, which is not realistic in the actual scenario. Since eavesdroppers may keep silent to hide themselves, the relay may have difficulty obtaining the accurate CSI between relay and eavesdropper, leading performance of the above algorithms to deterioration. Therefore, how to design a robust beamforming algorithm with tolerance for CSI disturbance to improve the security of SWIPT-assisted communication system is challenging work. Supposing CSI error follows Gaussian distribution, the optimal security rate is achieved by the joint optimization of relay beamformer and power allocation factors [13]. Assuming the CSI error between the base station and the user in a bounded ellipsoid region, a robust beamforming scheme based on S-procedure is proposed to achieve the minimum base station transmission power [14]. The robust sum rate optimization in non-orthogonal multiple access (NOMA) amplifying and forwarding (AF) relay networks is considered in [15], and the power allocation scheme and beamforming matrix are jointly optimized to achieve the maximum rate at the quality of service (QoS) constraints and relay transmission power constraints. In addition, a joint information beamforming and energy beamforming scheme based on semidefinite relaxation (SDR) and successive convex approximation (SCA) techniques is proposed to achieve the maximum secrecy rate of the multiple input single output (MISO) network [16], in which the CSI error is also restrained in a bounded elliptic region.

It is worth noting that the difference between our work and the above works are the following. Different from [13], we discussed the bounded ellipsoid model of the channel error instead of the Gaussian distribution model, which is widely used in the literature [14,15,16,17,18]. Although the bounded ellipsoid channel error model is discussed in [14,15,16,17,18], the point to point algorithm proposed in [14,16,17] can not be directly generalized to our SWIPT relay network. The security problem of two-tier downlink heterogeneous network is studied in [18], since channel error between the base station and the eavesdropper is not involved in [18], and their system models are too different from ours, and its conclusions could not be directly extended to SWIPT WSN. The research in [15] only focuses on how to obtain the best quality of service for users, while ignoring the problem of user information leakage. Our research can effectively prevent information leakage while maximizing user transmission rate.

The main contributions of our work are summarized as follows:

• We propose the robust optimization method to maximize the security rate of the destination under the constraints such as transmission power of relay, signal to interference plus noise power ratio (SINR) of receiver, and CSI error between relay and eavesdroppers. Our method solves the problem that the performance of existing methods deteriorates sharply when the relay fails to obtain perfect CSI. Since perfect CSI is difficult to obtain in practical scenarios, our method has better performance.
• To circumvent the non-convexity of the original optimization problem, the original problem is decoupled into two subproblems by introducing a slack variable, and thus can be iteratively solved by an SDR technique and bisection method, respectively.
• For simplicity, the scene without a direct connection between the source, the legitimate destination and the eavesdropper are mainly involved in the proposed algorithm; however, we have proved that the proposed algorithm can be generalized to SWIPT WSN with a direct connection between the source, the legitimate destination and the existing eavesdropper.
• Extensive numerical results are presented to demonstrate the system parameters impacting the performance of the proposed scheme and the superior performance of the proposed scheme against three other benchmark schemes.

The rest of this paper is organized as follows: the system model and the problem formulation are presented in Section 2. Then, we design an effective iterative algorithm to achieve the maximum secrecy rate in the worst case in Section 3. Section 4 presents numerical results to validate the proposed scheme. Finally, Section 5 concludes the paper and extends the future research work.

**Notations:** Bold lowercase letters and capital letters represent column vectors and matrices, respectively, The transpose, conjugate transpose, trace, and rank of a matrix S are denoted by $S^{T}$, $S^{H}$, $\mathrm{Tr}\left(S\right)$, and $\mathrm{rank}\left(S\right)$, respectively. ${\left[S\right]}_{i,j}$ signals the $\left(i,j\right)$ term of the matrix S. The modulus of a complex scalar *s* and euclidean norm of a vector s are denoted by $\left|s\right|$ and $∥s∥$, respectively. $I_{N}$ denotes the N-dimensional identity matrix. $\mathrm{diag}\left(s\right)$ is the diagonal matrix whose diagonal elements are the vector s. E[] representing mathematical expectation. ${\left[x\right]}^{+}$ is the abbreviation of $\max\left[x,0\right]$.

## 2. System Model and Problem Formulation

**Figure 1:**
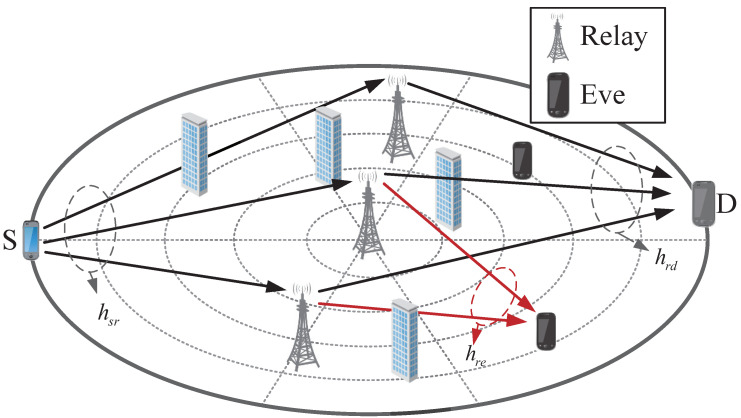
The SWIPT-assisted relay communication network model.

Consider the SWIPT-assisted relay communications system shown in Figure 1, which consists of a source (S), a legitimate destination (D), K eavesdroppers (Eve), and N energy limited relays (R). Each piece of the equipment is equipped with a single antenna. Furthermore, we assume that there is no direct link among S, D, and Eve (The algorithm proposed by this paper is also applicable to the case of direct link. Refer to Remark 1 at the end of Section 3 [19]), which usually occurs at the long-distance path loss or heavy channel fading [20,21,22,23,24,25]. For example, in a long range missile detection system, the satellite can act as a relay to forward information to the control center without a direct link between monitoring nodes. Here, we consider the fact that relay uses the power splitting (PS) scheme to harvest energy from the Radio Frequency (RF) signal transmitted by S to forward the confidential information to D in the meantime (There are two well-known practical receiver schemes (i.e., time switching (TS) and power splitting PS). PS scheme can process information and harvest energy at the same time to avoid interruption of information transmission, so we adopt PS scheme).

Following by SWIPT-AF relay protocol, relay employs cascading two stages to process the signal received by *i*-th relay [7]. In the first stage, namely the receiving stage, the signal received by *i*-th relay can be expressed as

(1) $$y_{r_{i}}=h_{sr_{i}}x_{s}+n_{a,i},\forall i,$$

where *x* represents the transmit signal at S with power $E\left({\left|x_{s}\right|}^{2}\right)=P_{s}$, $P_{s}$ represents the transmitted power of S. $h_{sr_{i}}$ represents the channel complex coefficient from S to *i*-th R, and $n_{a,i}∼\mathrm{CN}\left(0,\sigma_{a,i}^{2}\right)$ is the additive white Gaussian noise with 0 mean and $\sigma_{a,i}^{2}$ variance received by the *k*-TH relay.

**Figure 2:**
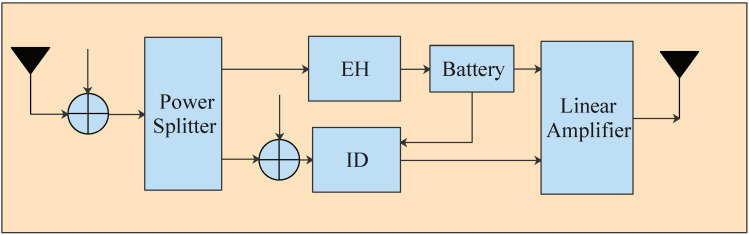
Power splitting receiver architecture.

Each AF relay adopts a PS receiver shown in Figure 2 to process signals. Specifically, the relay divides the received signal into two parts. One part $\left(\sqrt{1-\alpha_{i}}y_{r_{i}}\right)$ is used for information decoding (ID); the other part $\left(\sqrt{\alpha_{i}}y_{r_{i}}\right)$ is used for energy harvest, where $\alpha_{i}$ is the power splitting ratio. In general, the energy harvested from the received RF power may be modeled by a nonlinear equation. However, since there is currently no widely accepted nonlinear model related to energy harvest, we employ the linear energy collection model here [26]. Thus, the energy harvested by the relay is modeled as

(2) $$E_{R}=\eta \alpha P_{s}{∥h_{sr}∥}_{2}^{2},$$

where η(0<η<1) represents the EH efficiency and $h_{sr}={\left[h_{sr_{i}}\right]}_{i=1}^{N}$.

In the second stage, called the amplification and forwarding stage, linear amplification of the signal received by the *i*-th relay can be described as

(3) $$x_{r_{i}}=\beta_{i}\left(\sqrt{1-\alpha_{i}}y_{r_{i}}+n_{c,i}\right),\forall i,$$

where $n_{c,i}∼\mathrm{CN}\left(0,\sigma_{c}^{2}\right)$ is baseband conversion noise of the RF signal. $\beta_{i}$ is a complex coefficient given by

(4) $$\beta_{i}=\sqrt{\frac{\eta \alpha_{i}{\left|h_{sr_{i}}\right|}^{2}P_{s}}{\left(1-\alpha_{i}\right){\left|h_{sr_{i}}\right|}^{2}P_{s}+\left(1-\alpha_{i}\right)\sigma_{a}^{2}+\sigma_{c}^{2}}}e^{j\theta_{i}}.$$

Due to limited energy in the relay, the energy of the relay output signal must be less than the energy harvested by the relay, viz., the following constraints must be satisfied:

(5) $${\left|x_{r_{i}}\right|}^{2}⩽E_{R}.$$

To sum up, transmitting signals of relays can be described as

(6) $$x_{r}=Q_{\beta \alpha }h_{sr}\sqrt{P_{s}}x_{s}+Q_{\beta \alpha }n_{a}+Q_{\beta }n_{c},$$

where $Q_{\beta \alpha }$ and $Q_{\beta }$ are the diagonal matrix with diagonal elements are $(\beta_{1}\sqrt{1-\alpha_{1}},\ldots ,$ $\beta_{N}\sqrt{1-\alpha_{N}}{)}^{T}$ and ${\left(\beta_{1},\ldots ,\beta_{N}\right)}^{T}$, respectively. $n_{a}∼\mathrm{CN}\left(0,\sigma_{a}^{2}\right)$ is the additive white Gaussian noise received by R, where $\sigma_{a}^{2}$ represents the variance of $n_{a}$. $n_{c}∼\mathrm{CN}\left(0,\sigma_{c}^{2}\right)$ is the baseband conversion noise of the RF signal, where $\sigma_{c}^{2}$ represents the variance of $n_{c}$. Thus, signals received by the legitimate destination can be described as

(7) $$y_{d}=h_{rd}^{H}x_{r}+n_{d},$$

where $n_{d}∼\mathrm{CN}\left(0,\sigma_{d}^{2}\right)$ is the additive white Gaussian noise received by D, where $\sigma_{d}^{2}$ represents the variance of $n_{d}$. $h_{rd}={\left[h_{r_{i}d}\right]}_{i=1}^{N}$ represents the channel complex coefficient from relay to legitimate destination. Substituting (6) into (7), we can obtain

(8) $$y_{d}=h_{rd}^{H}Q_{\beta \alpha }h_{sr}\sqrt{P_{s}}x_{s}+h_{rd}^{H}Q_{\beta \alpha }n_{a}+h_{rd}^{H}Q_{\beta }n_{c}+n_{d}.$$

Meanwhile, the signal received by each eavesdropper is expressed as

(9) $$y_{e,k}={\bar{h}}_{re,k}^{H}x_{r}+n_{e,k},$$

where ${\bar{h}}_{re,k}$ represents the nominal channel complex coefficient from relay to *k*-th eavesdroppers, $n_{e,k}∼\mathrm{CN}\left(0,\sigma_{e,k}^{2}\right)$ is the additive white Gaussian noise. Substituting (6) into (9), the signal received by the *k*-th eavesdropper can be described as

(10) $$y_{e,k}={\bar{h}}_{re,k}^{H}Q_{\beta \alpha }h_{sr}\sqrt{P_{s}}x_{s}+{\bar{h}}_{re,k}^{H}Q_{\beta \alpha }n_{a}+{\bar{h}}_{re,k}^{H}Q_{\beta }n_{c}+n_{e,k}.$$

In summary, the SINR of D and Eve can be respectively described as

(11) $$\mathrm{SINR}{}_{S,D}=\frac{P_{s}{\left|h_{rd}^{H}Q_{\beta \alpha }h_{sr}\right|}^{2}}{\sigma_{a}^{2}{∥h_{rd}^{H}Q_{\beta \alpha }∥}^{2}+\sigma_{c}^{2}{∥h_{rd}^{H}Q_{\beta }∥}^{2}+\sigma_{d}^{2}},$$

(12) $$\mathrm{SINR}{}_{S,E,k}=\frac{P_{s}{\left|{\bar{h}}_{re,k}^{H}Q_{\beta \alpha }h_{sr}\right|}^{2}}{\sigma_{a}^{2}{∥{\bar{h}}_{re,k}^{H}Q_{\beta \alpha }∥}^{2}+\sigma_{c}^{2}{∥{\bar{h}}_{re,k}^{H}Q_{\beta }∥}^{2}+\sigma_{e,k}^{2}}.$$

Supposing CSI disturbance between R and Eve following the elliptical sphere uncertainty model, i.e.,

(13) $$h_{re,k}={\bar{h}}_{re,k}+\Delta h_{re,k},$$

where $h_{re,k}$ represents the actual channel coefficient vector between R and the *k*-th Eve, and $\Delta h_{re,k}$ represents the bounded estimate error described by $\Delta =\{\Delta h_{re,k}|$ ${∥\Delta h_{re,k}∥}^{2}⩽\delta^{2},\forall k,\delta ⩾0\}$, where δ is the upper bound on the error. It can be considered that the relay has perfect CSI when $h_{re,k}={\bar{h}}_{re,k}$. After introducing the channel error model, the SINR of the received signal of the *k*-th eavesdropper can be re-expressed as

(14) $$\mathrm{SINR}{}_{S,E,k}=\frac{P_{s}{\left|{\left({\bar{h}}_{re,k}+\Delta h_{re,k}\right)}^{H}Q_{\beta \alpha }h_{sr}\right|}^{2}}{\sigma_{a}^{2}{∥{\left({\bar{h}}_{re,k}+\Delta h_{re,k}\right)}^{H}Q_{\beta \alpha }∥}^{2}+\sigma_{c}^{2}{∥{\left({\bar{h}}_{re,k}+\Delta h_{re,k}\right)}^{H}Q_{\beta }∥}^{2}+\sigma_{e,k}^{2}}.$$

Hereto, we have obtained the SINR of the signals received by the D and the Eve, respectively, and we defined the secrecy rate as

(15) $$r_{Sec}=\min_{k=1\cdots K}{\left[r_{S,D}-r_{S,E,k}\right]}^{+},$$

where $r_{S,D}=1/2\log\left(1+\mathrm{SIN}R_{S,D}\right)$ represents the mutual information between S and D. $r_{S,E,k}=1/2\log\left(1+\mathrm{SIN}R_{S,E,k}\right)$ represents the mutual information between the *k*-th Eve and S. The coefficient 1/2 is due to the relay working in a half-duplex model.

For convenience, we may convert $r_{S,D}$ and $r_{S,E,k}$ into the following forms:

(16) $$\left\{\begin{array}{c} r_{S,D}=\frac{1}{2}\log_{2}\left(1+\frac{{\left|h_{sd}^{H}w\right|}^{2}}{{\left|g_{sd}^{H}w\right|}^{2}+\sigma_{d}^{2}}\right)\\ r_{S,E,k}=\frac{1}{2}\log_{2}\left(1+\frac{{\left|h_{se,k}^{H}w\right|}^{2}}{{\left|g_{\mathrm{se},k}^{H}w\right|}^{2}+\sigma_{e,k}^{2}}\right) \end{array}\right.,$$

where $w={\left[w_{1},w_{2},...,w_{N}\right]}^{T}$ is defined as a beamforming vector, and the other parameters are defined as follows:

(17) $${\left[h_{sd}\right]}_{i}\overset{\Delta }{\;=}h_{sr_{i}}h_{r_{i}d}\sqrt{\frac{\eta \alpha_{i}\left(1-\alpha_{i}\right)P_{s}}{\left(1-\alpha_{i}\right)\left({\left|h_{sr_{i}}\right|}^{2}P_{s}+\sigma_{a}^{2}\right)+\sigma_{c}^{2}}},\forall i;$$

(18) $${\left[g_{sd}\right]}_{i}\overset{\Delta }{\;=}h_{sr_{i}}h_{r_{i}d}\sqrt{\frac{\eta \alpha_{i}P_{s}\left(\left(1-\alpha_{i}\right)\sigma_{a}^{2}+\sigma_{c}^{2}\right)}{\left(1-\alpha_{i}\right)\left({\left|h_{sr_{i}}\right|}^{2}P_{s}+\sigma_{a}^{2}\right)+\sigma_{c}^{2}}},\forall i;$$

(19) $${\left[h_{se,k}\right]}_{i}\overset{\Delta }{\;=}h_{sr_{i}}h_{r_{i}e,k}\sqrt{\frac{\eta \alpha_{i}\left(1-\alpha_{i}\right)P_{s}}{\left(1-\alpha_{i}\right)\left({\left|h_{sr_{i}}\right|}^{2}P_{s}+\sigma_{a}^{2}\right)+\sigma_{c}^{2}}},\forall i,\forall k;$$

(20) $${\left[g_{se,k}\right]}_{i}\overset{\Delta }{\;=}h_{sr_{i}}h_{r_{i}e,k}\sqrt{\frac{\eta {\bar{\alpha }}_{i}P_{s}\left(\left(1-\alpha_{i}\right)\sigma_{a}^{2}+\sigma_{c}^{2}\right)}{\left(1-\alpha_{i}\right)\left({\left|h_{sr_{i}}\right|}^{2}P_{s}+\sigma_{a}^{2}\right)+\sigma_{c}^{2}}},\forall i,\forall k.$$

Considering that the error of CSI can be described by an elliptic bounded model, we intend to design a beamforming vector which may achieve the maximum secrecy rate in all possible eavesdropper CSI errors; thus, the optimization problem mentioned above can be described as

(21) maxwminΔhre,k∈Δ12rS,D−rS,E,k+s.t.TrηαiPshsri2wwHEi⩽ηαiPshsri2,∀iΔ={Δhre,k|Δhre,k2⩽δ2,∀k},

where $E_{i}=\mathrm{diag}\left(e_{i}\right)$, $e_{i}$ is the vector whose *i*-th entry is 1, and the other entries are 0. Considering that the channel error only exists between the eavesdropper and the relay, (21) can be translated into

(22) $$\begin{array}{c} \begin{array}{cc} \max_{w,\Delta h_{re,k}\in \Delta } & \frac{1}{2}\left(r_{S,D}-\max r_{S,E,k}\right)\\ \;s.t. & \mathrm{Tr}\left(\left(\eta {\bar{\alpha }}_{i}P_{s}{\left|h_{sr_{i}}\right|}^{2}ww^{H}\right)E_{i}\right)⩽\eta {\bar{\alpha }}_{i}P_{s}{\left|h_{sr_{i}}\right|}^{2},\forall i\\ & \Delta =\{\Delta h_{re,k}|{∥\Delta h_{re,k}∥}^{2}⩽\delta^{2},\forall k\}, \end{array} \end{array}$$

Observing (22), we can find that two optimized variables are coupled in objective functions; thus, the original problem is non-convex and hard to handle. We will explore the solution method of this problem in the next section.

## 3. Robust Beamforming Optimization for Physical Layer Security

In this section, we aim to settle the problem of maximizing the secrecy rate under the bounded CSI error model proposed in Section 2. To settle the above non-convex problem, we introduce a slack variable τ to convert the original problem into P1:

(23) $$\left(P1\right)\;\min_{\tau ,w}\;\frac{1}{2}r_{S,D}-\frac{1}{2}\log\left(\frac{1}{\tau }\right)$$

(24) $$\;\;\;s.t.\max_{\Delta h_{re,k}\in \Delta }r_{S,E,k}⩽\log\left(\frac{1}{\tau }\right),\forall k$$

(25) $$\;\;\;\;\;\;\;\mathrm{Tr}\left(\left(\eta \alpha_{i}P_{s}{\left|h_{sr_{i}}\right|}^{2}ww^{H}\right)E_{i}\right)⩽\eta \alpha_{i}P_{s}{\left|h_{sr_{i}}\right|}^{2},\forall i,$$

Observing (P1), it can be seen that, although the introduced variable τ solves the maximum and minimum forms of the source problem (22), due to the coupling of τ and w in the objective function (23) and the form of constraint (24), the problem (23) is still non-convex and unsolvable. To settle this problem, we propose an algorithm to transform it into a two-level optimization problem. Specifically, the lower-level optimization problem can be expressed as the following form (P2):

(26) $$\left(P2\right)\;\min_{w}\;\frac{{\left|h_{sd}^{H}w\right|}^{2}}{{\left|g_{sd}^{H}w\right|}^{2}+\sigma_{d}^{2}}$$

(27) $$\;\;\;\;s.t.\max_{{\Delta h}_{re,k}\in \Delta }r_{S,E,k}⩽\log\left(\frac{1}{\tau }\right),\forall k$$

(28) $$\;\;\;\;\;\;\;\;\mathrm{Tr}\left(\left(\eta \alpha_{i}P_{s}{\left|h_{sr_{i}}\right|}^{2}ww^{H}\right)E_{i}\right)⩽\eta \alpha_{i}P_{s}{\left|h_{sr_{i}}\right|}^{2},\forall i.$$

which can be regarded as a quadratic programming problem for the variable w when the variable τ is fixed.

The upper-level optimization problem is determined by the solution of the lower-level optimization problem. Let R(τ)=τγ(τ), where γ(τ) represents the objective function of the optimization problem (P2). The objective function of the optimization problem (23) could be modeled as

(29) $$\frac{1}{2}\log\left(1+\gamma \left(\tau \right)\right)-\frac{1}{2}\log\left(\frac{1}{\tau }\right)=\frac{1}{2}\log\left(\tau +R\left(\tau \right)\right).$$

Consequently, we can describe the upper-level optimization problem (P3) as

(30) $$\begin{array}{ccc} \left(P3\right) & \max_{\tau } & \frac{1}{2}\log\left(\tau +R\left(\tau \right)\right)\\ & s.t. & \tau_{\min}⩽\tau ⩽1, \end{array}$$

The meaning of 1/τ in problem (23) is the maximum allowable SINR of channels between relay and eavesdroppers which satisfies the non-zero security rate. $\tau_{\min}$ represents the smallest feasible value of τ given by the following inequality:

(31) $$\begin{array}{c} \tau ⩾\frac{1}{1+P_{s}{∥h_{sd}∥}^{2}{∥w∥}^{2}/\sigma_{d}^{2}}\\ \;⩾\frac{1}{1+NP_{s}{∥h_{sd}∥}^{2}/\sigma_{d}^{2}}\overset{\Delta }{\;=}\tau_{\min}, \end{array}$$

where the first inequality is obtained according to Cauchy–Schwarz inequality, and the second inequality is obtained by ${\left|w_{i}\right|}^{2}⩽1,\forall i$.

Suppose that we can obtain the corresponding R(τ) for any τ in the definition domain, and then the optimal solution $\tau^{*}$ of the (30) can be efficiently solved within the region $\left[\tau_{\min},1\right]$ through bisection method, etc. Heretofore, we have solved the upper-level problem (30), but, due to the non-convexity of constraint (27), the lower-level problem (26) is still difficult to settle. It is obvious that constraint (27) can be rewritten in the following form:

(32) $$\forall \Delta h_{re,k}\in \Delta \Rightarrow \frac{{\left|h_{se,k}^{H}w\right|}^{2}}{{\left|g_{\mathrm{se},k}^{H}w\right|}^{2}+\sigma_{e,k}^{2}}⩽\frac{1}{\tau }-1,$$

Considering the ellipsoidal uncertainty model, using S-procedure [27], inequation (32) can be expressed in the form of a series of linear matrix inequalities (LMI), which are summarized as the following proposition:

**Proposition** **1.** *The constraints (32) hold if and only if there exist $\mu_{k}⩾0,k=1,...K$ such that the following LMIs hold:*

(33) $$\left[\begin{array}{cc} \mu_{k}I_{N}+W & {W\bar{h}}_{re,k}^{H}\\ {\bar{h}}_{re,k}W & \psi_{k} \end{array}\right]⪰0,\forall k.$$

**Proof.**  See Appendix A.    □

Using Proposition 1, the constraint (27) can be converted to an LMI form, and then problem (26) can be expressed as

(34) $$\begin{array}{cc} \max_{W,\mu_{k},\psi_{k}} & \frac{\mathrm{Tr}\left({\hat{h}}_{rd}^{H}W{\hat{h}}_{rd}\right)}{\mathrm{Tr}\left({\hat{g}}_{rd}^{H}W{\hat{g}}_{rd}\right)+\sigma_{d}^{2}}\\ s.t. & W⪰0,\mu_{k}⩾0,\psi_{k}⩾0\\ & \left[\begin{array}{cc} \mu_{k}I_{N}+W & W{\bar{h}}_{re,k}\\ {\bar{h}}_{re,k}^{H}W & \psi_{k} \end{array}\right]⪰0,\forall k\\ & \mathrm{Tr}\left(\left(\eta \alpha_{i}P_{s}{\left|h_{sr_{i}}\right|}^{2}W\right)E_{i}\right)⩽\eta \alpha_{i}P_{s}{\left|h_{sr_{i}}\right|}^{2},\forall i, \end{array}$$

where ${\hat{h}}_{rd}\overset{\Delta }{\;=}\mathrm{diag}\left(1-\alpha_{i}\right)h_{rd}$, ${\hat{g}}_{rd}\overset{\Delta }{\;=}\mathrm{diag}\left(\left(1-\alpha_{i}\right)\sigma_{a}^{2}+\sigma_{c}^{2}\right)h_{rd}$.

At this point, the problem is easy to solve while still not a standard convex optimization problem. In order to settle the fractional form of the above problem, we define $\tilde{W}\;=\;\xi W$, $\xi \;=\;{\left(\mathrm{Tr}\left({\hat{g}}_{rd}^{H}W{\hat{g}}_{rd}\right)+\sigma_{d}^{2}\right)}^{-1}$ according to the Charnes–Cooper transformation. By combining the above definitions, we could convert the above problem into a standard convex optimization problem:

(35) $$\begin{array}{ccc} \left(P4\right) & \max_{\tilde{W},\mu_{k},\psi_{k}} & \mathrm{Tr}\left({\hat{h}}_{rd}^{H}\tilde{W}{\hat{h}}_{rd}\right)\\ & s.t. & \tilde{W}⪰0,\mu_{k}⩾0,\psi_{k}⩾0\\ & & \mathrm{Tr}\left({\hat{g}}_{rd}^{H}\tilde{W}{\hat{g}}_{rd}\right)+\sigma_{d}^{2}=\tau \\ & & \left[\begin{array}{cc} \mu_{k}I_{N}+\tilde{W} & \tilde{W}{\bar{h}}_{re,k}\\ {\bar{h}}_{re,k}^{H}\tilde{W} & \psi_{k} \end{array}\right]⪰0,\forall k\\ & & \mathrm{Tr}\left(\left(\eta \alpha_{i}P_{s}{\left|h_{sr_{i}}\right|}^{2}\tilde{W}\right)E_{i}\right)⩽\mu_{k}\eta \alpha_{i}P_{s}{\left|h_{sr_{i}}\right|}^{2},\forall i. \end{array}$$

Thus far, we convert the original non-convex problem (22) into two convex problems (P3) and (P4). (P3) can be solved by using the bisection method, and (P4) can be solved by using the interior point method or a convex optimization toolbox such as CVX [27]. If the solution ${\tilde{W}}^{*}$ of the problem (P4) satisfies $\mathrm{Rank}\left({\tilde{W}}^{*}\right)=1$, w could be obtained by EVD, otherwise by Gaussian randomization. In addition, the specific algorithm is summarized in Algorithm 1.
**Algorithm 1** Proposed algorithm for solving P1**1.** Initialize relevant basic parameters;**2.** Set $s=\tau_{\min},v=\tau_{\max}=1$, accuracy ϵ>0;**Repeat****3.**τcur=s+vl+u22;**4.** Solve (35), get $R\left(\tau_{cur}\right)$ and $\tilde{W}$;**5.** Solve (30), get $r_{cur}=\left(1/2\right)\log_{2}\left(\tau_{cur}+R\left(\tau_{cur}\right)\right)$;**6.** Set $\tau_{tem}=\max\left(\tau_{cur}-\Delta \tau ,\tau_{\min}\right)$, where Δτ>0 denotes a minimum,return $r_{tem}=(1/2)\log_{2}\left(\tau_{tem}+R\left(\tau_{tem}\right)\right)$;**7.** If $r_{cur}>r_{tem}$, $s=\tau_{cur}$,else $v=\tau_{cur}$;**8. until**$|r_{cur}-r_{tem}|<\epsilon$**9.**$W^{*}=\tilde{W}$, get $w^{*}$ by EVD or Gaussian randomization to $W^{*}$, $r^{*}=r_{cur}$.

**Remark** **1.** *Although the problem described in (22) is modeled without a direct link between S, D, and Eve, it is can be easily extended to the case direct link between S, D, and Eve existing. Let a direct link channel coefficient between S and D be defined as $h_{sd-DL}$ and the direct link channel coefficient vector between S and the k-th Eve be defined as ${\bar{h}}_{se,k-DL}$, respectively. The signals received by D and Eve through the direct link could be respectively defined as*

(36) $$y_{S,D-DL}=h_{sd-\mathrm{DL}}x_{s}+n_{sd-DL},$$

(37) $$y_{S,E,k-DL}={\bar{h}}_{se,k-\mathrm{DL}}x_{s}+n_{se,k-DL}.$$

*where $n_{sd-DL}∼\mathrm{CN}\left(0,1\right)$ represents the noise signal received by D, $n_{se,k-DL}∼\mathrm{CN}\left(0,1\right)$ represents the noise signal received by Eve.* *By stacking the direct link signals and relay forward signals received by the D and Eve into a 2×1 vector, respectively, the reception rate of D and Eve can be defined as*

(38) $$\begin{array}{c} r_{S,D-\mathrm{DL}}=\log\left(1+P_{s}{\left|h_{sd-\mathrm{DL}}\right|}^{2}+\frac{{\left|h_{sd}^{H}w\right|}^{2}}{{\left|g_{sd}^{H}w\right|}^{2}+\sigma_{d}^{2}}\right),\\ r_{S,E,k-\mathrm{DL}}=\log\left(1+P_{s}{∥{\bar{h}}_{\mathrm{se},k-\mathrm{DL}}∥}_{2}^{2}+\frac{{\left|h_{se,k}^{H}w\right|}^{2}}{{\left|g_{\mathrm{se},k}^{H}w\right|}^{2}+\sigma_{e}^{2}}\right). \end{array}$$

*It can be seen that the only difference between (38) and (16) is the additive constant. Similar to a model without a direct link between the S, D, and eavesdropper, supposing a direct link between existing D and S, imperfect CSI between relay and eavesdropper can also be modeled as a bounded ellipsoid, i.e., ${∥h_{se,k-DL}-{\bar{h}}_{se,k-DL}∥}_{2}⩽\xi_{k}$, where $\xi_{k}$ represents the upper bound of channel error of the k-th eavesdropper. Then, the worst-case transmission rate of system is described as*

(39) maxhse,k−DL:hse,k−DL−h¯se,k−DL2⩽ξkrS,E,k−DL=log1+Psh¯se,k−DL+ξk2+hse,kHw2gse,kHw2+σe2,

*which again differs from the no-direct link term in (16) only in some constant term [21]. Hence, our model of direct link non-existing can be straightforwardly extended to the case of direct link existing. However, it is worth noting that, if the direct links between S and Eve are strong, a positive secrecy rate may be achieved with difficulty.*

## 4. Complexity Analysis and Numerical Results

In this section, we first derive the complexity of the proposed algorithm. By analyzing Algorithm 1, it can be found that the complexity of the proposed algorithm is concentrated from solved (P2) in each iteration. Due to the LMI form introduced by S-procedure and SDR, the complexity of the problem in (P2) is mainly derived from solving the following quantitative LMI including *K* linear matrix inequalities of N+1 dimensions, *N* linear matrix inequalities of 1 dimension, and *K* linear matrix inequalities of one dimension. Similar to [28], the complexity of the proposed robust secure beamforming algorithm can be roughly expressed as $\sqrt{K\left(N+1\right)}K^{2}N^{4}\ln\left(1/\varepsilon \right)$.

Next, we present extensive numerical simulation results to prove the performance of the proposed algorithm. Our comparison algorithm comes from similar works presented in [7,12,13]. As shown in Figure 1, we assume that the S is located at the edge of a circular region and the legitimate destination is located at a symmetrical position of the source on the circle. AF relay and eavesdroppers (only existing in the “security zone”) are randomly distributed within a circle of radius R. Similar to some related work, we assume that all channel models used in the paper contain large-scale path loss and small-scale multipath fading, the small-scale fading is assumed to be Rician fading, and the path loss model can be described as

$$L=A_{0}{\left(\frac{d}{d_{0}}\right)}^{-\kappa }.$$

Parameters related to experiments are shown in Table 1.

**Figure 3:**
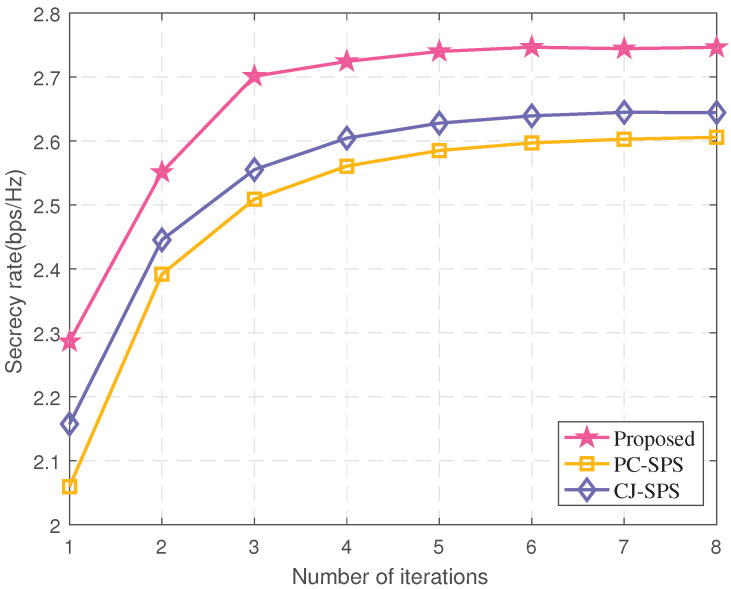
The secrecy rate vs. the number of iterations (*N* = 10, *K* = 5).

Convergences of the algorithms are plotted in Figure 3. It can be seen that the convergence of the three algorithms is similar, and all converge to the maximum within seven iterations, but the proposed algorithm achieves a higher secrecy rate. The reason is that the cooperative jamming beamforming (CJ-SPS) algorithm [7] only considers the performance in the case of perfect CSI. Although AN is introduced to interfere with eavesdroppers, the security of the system is still deteriorating in the presence of CSI errors. Since designs for CSI error follow Gaussian distribution, the probabilistic constraint beamforming (PC-SPS) algorithm [13] has a certain robustness, and its performance is slightly better than the CJ-SPS algorithm, while the proposed algorithm can achieve a higher security rate at the ellipsoidal bounded CSI error.

**Figure 4:**
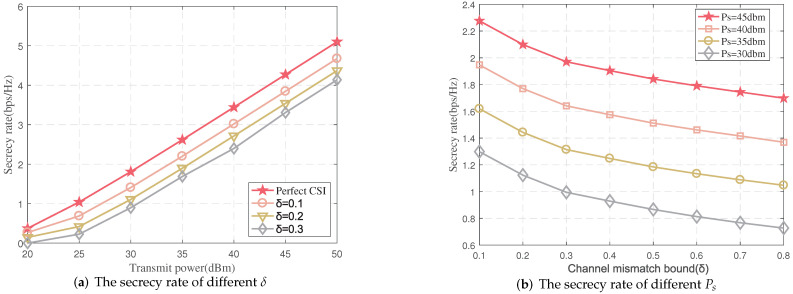
The secrecy rate vs. $P_{s}$ and δ (*N* = 10, *K* = 5).

In the proposed algorithm, the levels of CSI error impacting the security of the system are plotted in Figure 4a. As can be seen from the figure, the secrecy rate increases with the source transmission power increasing. It is mainly because of harvest energy of relay increasing with source transmission power, resulting in more transmission power of relay. Moreover, the security of system will decrease with CSI error increasing. In addition, Figure 4b shows that the secrecy rate varies with the channel error at the different transmission power of the source, and the conclusion is similar to Figure 4a. With the CSI error increasing, the achieved secrecy rate decreases gradually, and this is because the CSI has greater uncertainty within a larger error bound, leading the worst-case achieved secrecy rate to increases.

**Figure 5:**
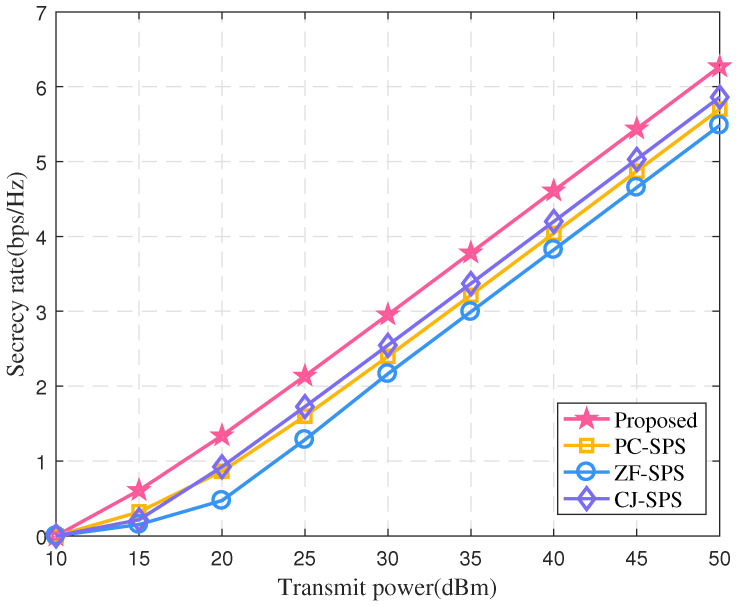
The secrecy rate vs. $P_{s}$ (*N* = 20, *K* = 8).

The performance of the proposed algorithm is compared with the three benchmark algorithms mentioned in the introduction in Figure 5. Clearly, relay may achieve higher secrecy rate with the transmitting power $P_{s}$ increasing. In addition, compared with the other benchmark algorithms, the performance of the proposed algorithm is improved as expected. It is worth noting that the PC-SPS algorithm models CSI error as Gaussian distribution, which is inconsistent with actual CSI error, thus leading to the worst performance.

**Figure 6:**
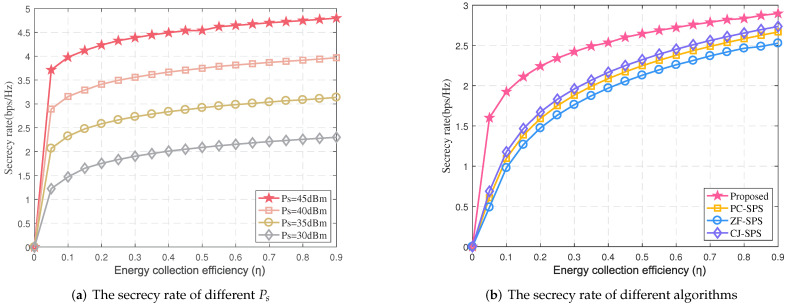
The secrecy rate vs. η (*N* = 15, *K* = 5).

Next, Figure 6a compares the system performance of our scheme at different energy collection efficiency η. As expected, the security rate increases with the energy collection efficiency η at any transmission power. The reason for the above phenomenon is that the relay can obtain more energy with the η increasing, thus having more transmission power. However, since SINR obtained by the eavesdropper may also enhance following η increasing, the increasing trend of security rate may slow down at the large η. The security rate of four algorithms at the different η are plotted in Figure 6b. Similar to Figure 6a, the secrecy rate of all algorithms increases with η. Moreover, our proposed algorithm achieves the highest secrecy rate since bounded CSI errors impacting the performance of the system are fully considered.

**Figure 7:**
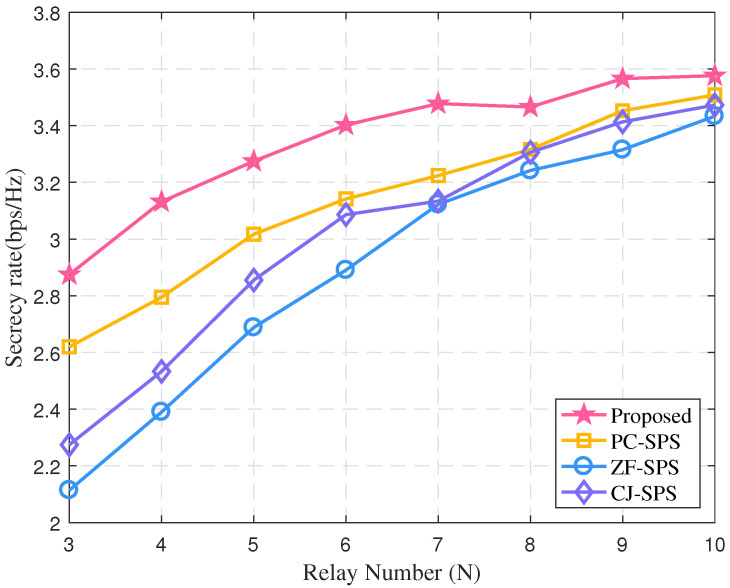
The secrecy rate vs. the number of relays (*K* = 1).

The number of relays impacting the security rate of system is described in Figure 7. It can be seen that security rate of all algorithms tends to increase with number of relays. This is of course because the ability of system may be enhanced with the number of relays increasing. Moreover, since the bounded CSI error impacting the performance of the system is fully considered, the proposed algorithm achieves the highest security rate among all the algorithms.

**Figure 8:**
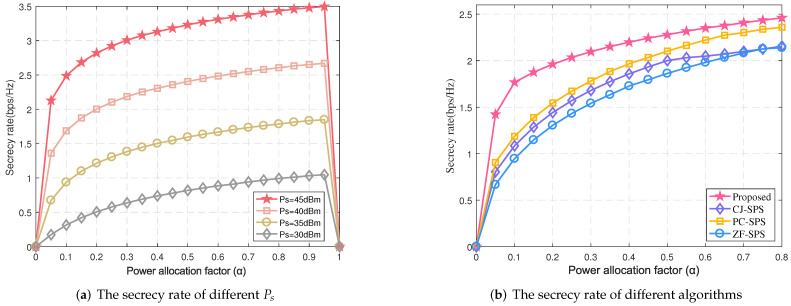
The secrecy rate vs. α (*N* = 10, *K* = 5).

Finally, the worst-case secrecy rates at the different power allocation factors are also described in Figure 8a. Similar to Figure 6, with the power allocation factor α increasing, the relay can obtain more energy, which leads to a higher secrecy rate. However, the secrecy rate drops sharply once α is greater than 0.9, since there is little signal to be forwarded. The security rate of four schemes at the different power allocation factor α are also compared in Figure 8b. Similar to Figure 8a, the secrecy rate of all algorithms increases with α. Moreover, our proposed algorithm achieves the highest secrecy rate since bounded CSI errors impacting the performance of the system are fully considered. It should be pointed out that, with the α increasing, the growth of the secrecy rate gradually tends to be flat, so using large α may be unadvisable and energy wasting.

## 5. Conclusions and Future Research

The robust beamforming design related to the secrecy rate of SWIPT WSN with relay is deeply discussed in this paper. Assuming bounded ellipsoid of CSI error between the relay and the eavesdropper, a maximum secrecy rate algorithm of the system at the constraints of relay power and bounded CSI error is proposed. By introducing slack variables, the original non-convex optimization is converted into a two-level coupled optimization problems. In addition, using S-procedure and SDR techniques, the non-convex constraints of lower-level problem are transformed into a series of LMI forms and iterative bisection methods are introduced to tackle the above decouple problem. The effectiveness of the proposed algorithm is verified by comparing it with PC-SPS, CJ-SPS, and ZF-SPS algorithms.

In this paper, we focus on the impact of channel errors on physical layer security, and we adopt a linear EH model at the relay. There are also many nonlinear EH models in the literature, and it is also necessary to study the influence of the nonlinear EH model on the network. However, because of the complexity of the nonlinear EH model, this is a challenging problem. In addition, the common relay cooperation technology is adopted in this paper, and the emerging intelligent reflecting surface technology is expected to replace relay in short-distance communication because of its excellent energy-saving effect. The SWIPT network of intelligent reflecting surface cooperation is also a subject of concern. We will also continue to pay attention to the above issues in the follow-up work.

## Figures and Tables

**Table 1:** Simulation parameter setup.

Paramates	Notation	Typical Value
Standard Distance	$d_{0}$	1m
Path loss exponent	κ	2.5
Channel mismatch bound	δ	0.1
Power splitting ratio	α	0.5
Fade coefficient	$A_{0}$	$10^{-3}$
Energy collection efficiency	η	50%
Circular area of radius R	*R*	50m
Power of source	$P_{s}$	30dBm
Noise variance of relay	$\sigma_{a}^{2}$	−80dBm
Noise variance of baseband	$\sigma_{c}^{2}$	−50dBm
Noise variance of receiver	$\sigma_{d}^{2}$	−130dBm
Noise variance of eavesdropper	$\sigma_{e,k}^{2}$	−130dBm

## Data Availability

The data presented in this study are available on request from the first author.

## Appendix A

**Proof** **of Proposition 1** Let $\tilde{w}=\sqrt{\frac{\eta \alpha P_{s}}{\left(1-\alpha \right)\left({\left|h_{sr}\right|}^{2}P_{s}+\sigma_{a}^{2}\right)+\sigma_{c}^{2}}}\mathrm{diag}(h_{sr})w$, The constraint (27) can be expressed as

(A1) $${∥\Delta h_{re,k}∥}^{2}⩽\delta^{2}$$

(A2) $${\left({\bar{h}}_{re,k}+\Delta h_{re,k}\right)}^{H}\tilde{w}{\tilde{w}}^{H}\left({\bar{h}}_{re,k}+\Delta h_{re,k}\right)+\frac{\sigma_{e,k}^{2}}{\left(1-\alpha \right)\sigma_{a}^{2}+\sigma_{c}^{2}-\frac{1\;-\alpha }{1/\tau -1}}⩾0,\forall k.$$

Then, according to SDR, let $W=\tilde{w}{\tilde{w}}^{H}$, (A2) converts to

(A3) $${\left({\bar{h}}_{re,k}+\Delta h_{re,k}\right)}^{H}W\left({\bar{h}}_{re,k}+\Delta h_{re,k}\right)+\frac{\sigma_{e,k}^{2}}{\left(1-\alpha \right)\sigma_{a}^{2}+\sigma_{c}^{2}-\frac{1-\alpha }{1/\tau -1}}⩾0,\forall k.$$

**Lemma** **A1.**
*Let $Q_{1},Q_{2}\in C^{N\times N}$, $b_{1},b_{2}\in C^{N\times 1}$, $c_{1},c_{2}\in C$ and $x\in C^{N\times 1}$, and then the inequation xHQ1x+2Reb1Hx+c1⩽0⇒ xHQ2x+2Reb2Hx+c2⩽0 hold if and only if there exists a μ⩾0 such that*

(A4)
$$\mu \left[\begin{array}{cc} Q_{1} & b_{1}\\ b_{1}^{H} & c_{1} \end{array}\right]-\left[\begin{array}{cc} Q_{2} & b_{2}\\ b_{2}^{H} & c_{2} \end{array}\right]⪰0,$$

*provided there exists a point $\hat{x}$ with x^HQ1x^+2Reb1Hx^+c1⩽0 [4].*
By applying Lemma A1, let $x={\tilde{h}}_{re,k}$, $Q_{1}=I_{N}$, $b_{1}=0$, $c_{1}=-\delta^{2}$, $Q_{2}=-W$, $b_{2}=-W{\bar{h}}_{re,k}$, $c_{2}=-{\bar{h}}_{re,k}^{H}W{\bar{h}}_{re,k}-\frac{\sigma_{e}^{2}}{\left(1-\alpha \right)\sigma_{a}^{2}+\sigma_{c}^{2}-\frac{1\;-\;\alpha }{1/\tau -1}}$, the inequations (A1) and (A2) can be transformed into

(A5) $$\left[\begin{array}{cc} \mu_{k}I_{N}+W & W{\bar{h}}_{re,k}\\ {\bar{h}}_{re,k}^{H}W & \psi_{k} \end{array}\right]⪰0,\forall k,$$

where $\psi_{k}\overset{\Delta }{\;=}{\bar{h}}_{re,k}^{H}W{\bar{h}}_{re,k}+\frac{\sigma_{e}^{2}}{\left(1-\alpha \right)\sigma_{a}^{2}+\sigma_{c}^{2}-\frac{1-\alpha }{1/\tau -1}}-\delta^{2}$. □
